# Healthy lifestyle and its change attenuated the risk of hypertension among rural population: evidence from a prospective cohort study

**DOI:** 10.3389/fpubh.2025.1529570

**Published:** 2025-02-06

**Authors:** Tingyun Ren, Yinghao Yuchi, Wei Liao, Ning Kang, Ruiying Li, Chongjian Wang

**Affiliations:** Department of Epidemiology and Biostatistics, College of Public Health, Zhengzhou University, Zhengzhou, Henan, China

**Keywords:** healthy lifestyle, blood pressure, hypertension, lifestyle change, rural population

## Abstract

**Objectives:**

Lifestyle may potentially influence blood pressure level, but the association of multiple healthy lifestyles with hypertension was limited, especially for rural population. The study aimed to explore the relationship of healthy lifestyles on hypertension, and then whether lifestyle change could influence hypertension in rural adults.

**Methods:**

A total of 16,454 participants were enrolled from the Henan Rural Cohort study, in China. The healthy lifestyles score (HLS) was concluded by smoking status, alcohol consumption, physical activity, diet status and body mass index. Associations of HLS and lifestyle change with systolic blood pressure (SBP) and diastolic blood pressure (DBP) were analyzed by generalized linear models, and with hypertension were analyzed by logistic regression model and restricted cubic spline plots.

**Results:**

The results from the generalized linear models showed SBP and DBP levels decreased with the HLS increasing (*P_trend_* < 0.01). Compared with participants with lower HLS (scored 0–2), the odds ratios (*OR*) and 95% confidence intervals (*CIs*) for hypertension in those with HLS = 3, 4, or 5 were 0.853 (0.737, 0.987), 0.881 (0.754, 1.029), and 0.658 (0.519, 0.834), respectively. And compared with participants with unhealthy lifestyle consistently, those changing lifestyle from unhealthy to healthy had lower levels of blood pressure [*β* (95% *CI*): SBP: −1.603 (−2.539, −0.668). DBP: −1.713 (−2.326, −1.100)] and hypertension risk [*OR* (95%*CI*): 0.744 (0.594, 0.931)]. Similar results could be found by the sensitivity analysis.

**Conclusion:**

The findings showed that healthy lifestyles could reduce blood pressure and hypertension risk, and that implementing healthier lifestyle changes could be an effective strategy to prevent hypertension in rural area.

## 1 Introduction

Health inequality has become a major concern in recent times, especially for residents living in resource-limited districts, who were less likely to receive adequate medical resources and support, putting them at a higher risk of diseases (1). With advancements in infectious diseases control and improvements in living standards, chronic non-communicable diseases, such as hypertension, have become the leading causes of death (2). Hypertension, a major risk factor of cardiovascular diseases, remains the leading cause of global mortality and a substantial contributor to disability-adjusted life-year (3). The prevalence of hypertension among adults was higher in low- and middle-income countries (LMICs) (31.5%, 1.04 billion people) than that in high-income countries (28.5%, 349 million people) (4). Moreover, the global prevalence of hypertension has been rising, especially in LMICs (5). In China, hypertension ranged from nearly 19% in 2002 to 23.2% in 2015 among adults, which has become one of the most critical public health issues (6). Previous studies have reported a high prevalence of hypertension, coupled with low awareness, treatment, and control rates in rural areas of China (7). Thus, it is critical to prevent and control hypertension in Chinese rural areas.

The risk factors of hypertension included genetic factors, environmental influences, and lifestyles (4, 5). An Australian longitudinal study demonstrated that individuals with more high-risk lifestyle factors had a higher likelihood of developing hypertension (8). But limited research has focussed on the association between multiple lifestyles and hypertension in LMICs. Recently, the healthy lifestyle score (HLS) or healthy lifestyle index (HLI) have been developed to explore the association between multiple lifestyles and chronic diseases, as well as life expectancy (9, 10), which provided a scientific basis for the early prevention of hypertension. Previous research has reported that lifestyle changes are a mainspring of the increasing prevalence of hypertension (11), and that lifestyle interventions can be effective in preventing the condition (12). Regular physical activity (13, 14), maintaining a normal body weight (15) and good dietary habits (16, 17) are recommended as key healthy lifestyles in the prevention and management of hypertension. However, the evidence of the effect of lifestyle changes on hypertension and blood pressure measures in rural adults is limited.

Although some studies in LMICs have shown that lifestyle interventions can reduce hypertension rates in rural populations, research examine the impact of multiple lifestyles factors on hypertension and blood pressure measures are lacking. In the United States, the majority of chronic disease lifestyle intervention research in rural populations has been conducted with small samples and lacks a comprehensive approach. Existing programs have shown short-term benefits, but evidence on sustained impact and long-term effectiveness is limited (18). Addressing these gaps is critical to designing comprehensive lifestyle interventions for chronic disease management in remote areas on a global scale. Therefore, this study aimed to (1) examine the association of multiple lifestyles with blood pressure measures and hypertension, and (2) investigate whether lifestyle changes are associated with the incidence of hypertension in rural adults. It is hypothesized that a higher HLS will be associated with lower blood pressure levels and a reduced risk of hypertension. Additionally, it is expected that lifestyle improvements over time will contribute to a decreased incidence of hypertension in rural populations.

## 2 Methods

### 2.1 Study population and design

The population were selected from the general population participating in the Henan Rural Cohort study, established in five rural areas of Henan province, China, to investigate the prevalence of chronic diseases and associated risk factors. The cohort study initially enrolled 39,259 permanent residents aged 18–79 years through a multistage stratified cluster sampling method during 2015–2017 at baseline. More detailed information of this cohort study has been described elsewhere (19). After a mean follow-up duration of 3.83 years, 35,995 participants were interviewed during the first follow-up between 2018 and 2022, of which 26,030 participants were interviewed in person. For this analysis, participants with hypertension at baseline (*n* = 8,538) were excluded, as well as those with missing hypertension data (*n* = 676) and lifestyle data (*n* = 362) during the follow-up interview. The exclusion of individuals with incomplete hypertension or lifestyle data aimed to minimize bias and strengthen the reliability of the findings. Consequently, a total of 16,454 participants were included in the prospective cohort study (Supplementary Figure S1).

The Henan Rural Cohort study was approved by the Zhengzhou University Life Science Ethics Committee and conducted in accordance with the principles of the Declaration of Helsinki. Before the study commenced, written informed consents were signed by all participants.

### 2.2 Measurement of blood pressure indicators and definition of hypertension

A calibrated electronic sphygmomanometer (HEM-770A Fuzzy, Omron, Kyoto, Japan) was used to measure blood pressure three times in a sitting position according to the American Heart Association’s standardized protocol (20). Participants were asked to rest for at least 30 min before the measurement and to keep quiet during it. Each measurement was conducted with an interval of at least 30 s. Systolic blood pressure (SBP, mmHg) and diastolic blood pressure (DBP, mmHg) were recorded three times, and the average value of three measurements was calculated for analysis. According to the 2018 Chinese guidelines for the management of hypertension, hypertension was defined as having a measured SBP ≥140 mmHg and/or DBP ≥ 90 mmHg, or having a self-report of physician-diagnosed hypertension and anti-hypertension treatment in the last 2 weeks (21).

### 2.3 Definition of healthy lifestyle

The Healthy Lifestyle Score (HLS) was calculated by simply summing five key lifestyle factors: smoking status, alcohol consumption, physical activity, diet status, and body mass index (BMI), which were collected through face-to-face interviews. These factors reflect critical aspects of typical lifestyle patterns in rural populations, covering both behavioral and dietary practices (1, 2, 22). For smoking status, participants were asked, “Do you smoke now?,” with response options of “Never,” “Former,” or “Yes.” For former smokers, an additional question “Why did you quit smoking” were asked by interviewer. For alcohol consumption, the frequency and volume of alcohol intake, including white wine, beer, red wine and yellow wine, were recorded to compute the average daily alcohol consumption. For physical activity, the Chinese version of the International Physical Activity Questionnaire was used to collect the data about the frequency (days per week) and duration of vigorous physical activity, moderate physical activity, and walking, from which the metabolic equivalent was calculated (21). For diet status, a valid Chinese version of food frequency questionnaire (23) was used to assess the frequency of 12 conventional food groups, including staple food, red meat, white meat, fish, eggs, dairy food, fruits, vegetables, been products, nut, pickles, whole grains, and animal oil. The diet score was calculated based on the consumption of specific items such as red meat, fish, eggs, dairy food, fruits, vegetables, been products, nut, and whole grains. For BMI, the height and weight were measured twice by trained staff using standard tapes and V. BODY (HBF-371, OMRON, Japan). The average values were used for analysis. BMI (kg/m^2^) was calculated as weight (kg) divided by the square of height (m).

The criteria for healthy lifestyles were defined according to previous studies (24, 25). For each lifestyle factor, participants would gain a score of 1 if they met the definition of the healthy group, and 0 otherwise. For smoking status, participants who never smoked or quit smoking for reasons other than illness for at least 6 months were classified as the healthy group (24). To avoid misclassification bias, former smokers who quite due to illness or in past 6 months were included in the current smokers group (24). For alcohol consumption, the healthy group included individuals who either never consumed alcohol or drank in moderation [less than 25 g/day for men, less than 15 g/day for women according to the Chinese dietary guidelines (26)]. For physical activity, moderate and vigorous physical activity qualified participants as the healthy group (27). For diet status, the diet score was computed based on the valid Chinese version of FFQ (23), with a score ranged from 0 to 36, detailed in Supplementary Table S1. A score in the top 40% (≥22) indicated a healthy diet (25). The healthy BMI group included those with a BMI of 18.5–23.9 kg/m^2^, according to the standard classification of normal weight specific for Chinese (28). Description and prevalence of the components of healthy lifestyle score are presented in Supplementary Table S2.

The HLS was computed as the sum of these five lifestyle factors, ranging from 0 to 5 points, with higher scores indicating a healthier lifestyle. The simple summation approach has been widely adopted in studies involving rural populations, as it effectively captures the core lifestyle characteristics of these communities while ensuring practicality and feasibility during field surveys (24, 25). Additionally, the changes of lifestyle between baseline and follow-up were classified into four group: consistently low (HLS in both baseline survey and follow-up survey =0–2), low to high (HLS in baseline = 0–2, in follow-up =3–5), high to low (HLS in baseline = 3–5, in follow-up = 0–2), and consistently high (HLS in both baseline survey and follow-up survey = 3–5).

### 2.4 Covariates

Age (continuous variable), gender (men and women), married status (married/cohabiting and widowed/single/divorced/separated), educational level (elementary school or below, junior high school, and senior high school or above), per capita monthly income level (<500 RMB, 500- RMB or ≥1,000 RMB), and history of chronic diseases [including type 2 diabetes mellitus (T2DM), coronary heart disease (CHD) and stroke, yes and no] were collected by well-trained staff during the face-to-face interview. The selection of covariates in this study was guided by previous research (29, 30). It should also be noted that employment status was not included as a covariate in this study, as the majority of the rural population are farmers, adjusting for this variable less relevant. Additionally, healthcare access was not adjusted for, as most participants are covered by a unified rural healthcare system, thereby reducing variability.

### 2.5 Statistical analysis

The statistical description of continuous and categorical variables was presented as means ± standard deviation (SD) and frequency (percentages), respectively. *T*-test was performed to compare differences between different groups for continuous variables, while Chi-squared test was utilized for categorical variables. Participants with HLS of 0, 1, and 2 were combined into the low HLS group based to ensure sufficient statistical power and reduce potential bias due to small sample sizes in individual low-score groups.

The associations between the healthy lifestyle and blood pressure measures (SBP and DBP) were testified by generalized linear models, with results showed as *β* coefficients and 95% confidence intervals. The binary logistics regression models were examined the association between the healthy lifestyle and hypertension, with results reported by odds ratio (OR) and 95% CIs. Furthermore, the trend of increasing HLS with blood pressure measures and hypertension was examined by regarding HLS as the continuous variable into the analysis, with *p* values for trend calculated. The adjusted model included age, gender, educational level, married status, per capita monthly income level, and history of chronic diseases (including type 2 diabetes mellitus, coronary heart disease and stroke). And the restricted cubic spline plots were used to explore the dose–response relationships between HLS, blood pressure measures and hypertension. Besides, the associations between lifestyle changes and blood pressure measures and hypertension were analyzed to find out whether lifestyle changes could prevent hypertension.

There were five sensitivity analysis to evaluate the robustness of the main analysis: (1) Use the OR of the association with single lifestyle and hypertension as the coefficient to get the weighted HLS, and further explore the association of the weighted HLS with blood pressure measures and hypertension; (2) The adjusted model was further adjusted for region to examine potential regional effects on the results; (3) Exclude new hypertension case who had hypertension in 1 year after the baseline survey to reduce the misclassification bias; (4) Exclude participants with coronary heart disease or stroke in the baseline survey to mitigate the influence of medication use on blood pressure; (5) Analyze the association of HLS with blood pressure stratified by anti-hypertension medicine use among hypertension patients to avoid the influence of medication on blood pressure.

Data were analyzed using SPSS software version 21.0 and R software version 3.5.3. All *p*-values were two-tailed with a statistical significance level of 0.05.

## 3 Results

### 3.1 Characteristics of study participants

Table 1 shows the baseline characteristics of study participants, stratified by healthy lifestyle score. Participants with a healthier lifestyle were more likely to be women. And individuals with a higher lifestyle score were younger, more educated, had higher income levels, and were more likely to be married or cohabiting. They also exhibited a lower level of alcohol intake and BMI, engaged in higher levels of physical activity and diet equality, and had lower prevalence of current smoking, T2DM, CHD, stroke, and hypertension (all *p* < 0.05). Moreover, participants with a higher lifestyle have a lower level of SBP and DBP (all *p* < 0.05).

**Table 1:** Baseline characteristics of study participants classified by healthy lifestyle score (*n* = 16,454).

Characteristics	HLS			
	0–2	3	4	5
	(*n* = 3,346)	(*n* = 6,141)	(*n* = 5,449)	(*n* = 1,518)
Age (years), mean ± SD	56.3 ± 11.1	55.1 ± 11.1	53.5 ± 11.4	52.0 ± 12.4
Women, *n* (%)	948 (28.3)	3,944 (64.2)	4,269 (78.3)	1,275 (84.0)
Education level, *n* (%)				
Elementary school or below	1,471 (44.0)	2,801 (45.6)	2,297 (42.2)	530 (34.9)
Junior high school	1,429 (42.7)	2,564 (41.8)	2,301 (42.2)	716 (47.2)
Senior high school or above	446 (13.3)	776 (12.6)	851 (15.6)	272 (17.9)
Married/cohabiting, *n* (%)	3,056 (91.3)	5,606 (91.3)	5,016 (92.1)	1,422 (93.7)
Per capita monthly income, *n* (%)				
<500 RMB	1,252 (37.4)	2,230 (36.3)	1853 (34.0)	467 (30.7)
500- RMB	1,116 (33.4)	2088 (34.0)	1808 (33.2)	467 (30.8)
≥1,000 RMB	978 (29.2)	1823 (29.7)	1788 (32.8)	584 (38.5)
Current smoking, *n* (%)	1,654 (49.4)	1,144 (18.6)	322 (5.9)	0
T2DM, n (%)	295 (8.8)	441 (7.2)	279 (5.1)	64 (4.2)
CHD, *n* (%)	125 (3.7)	241 (3.9)	178 (3.3)	48 (3.2)
Stroke, *n* (%)	167 (5.0)	249 (4.1)	175 (3.2)	33 (2.2)
Alcohol intake (g/day), mean ± SD	47.2 ± 60.6	23.1 ± 40.9	11.6 ± 24.3	6.8 ± 6.4
PA (MET-hour/week), mean ± SD	96.3 ± 64.6	130.3 ± 66.9	145.7 ± 64.3	155.1 ± 61.4
Diet score, mean ± SD	18.4 ± 3.6	19.4 ± 4.0	21.5 ± 4.1	24.3 ± 2.1
BMI (kg/m^2^), mean ± SD	25.3 ± 3.4	24.9 ± 3.4	23.4 ± 3.0	21.9 ± 1.4
SBP (mmHg), mean ± SD	117.1 ± 11.2	116.1 ± 11.7	115.0 ± 11.9	112.9 ± 11.9
DBP (mmHg), mean ± SD	73.7 ± 8.0	72.7 ± 8.1	71.6 ± 8.0	70.1 ± 7.9

BMI, body mass index; CHD, coronary heart disease; DBP, diastolic blood pressure; HLS, healthy lifestyle score; MAP, mean artery pressure; MET, Metabolic equivalent; PA, physical activity; RMB, Renminbi; SBP, systolic blood pressure; SD, standard distance; T2DM, type 2 diabetes mellitus.

The statistical description of continuous and categorical variables was presented as means ± standard deviation (SD) and frequency (percentages), respectively.

*T-test and Chi-squared test were performed to compare differences between different groups for continuous variables and categorical variables, respectively.*

The description and prevalence of the components of healthy lifestyle score are presented in Supplementary Table S2. For all participants in the baseline, the highest percentage of healthy lifestyle was alcohol consumption (92.8%), followed by smoking status (75.9%), physical activity (71.8%), diet status (39.6%), and BMI (46.2%). And in the first follow-up, the highest percentage of healthy lifestyle was alcohol consumption (93.5%), followed by smoking status (76.6%), physical activity (63.7%), BMI (45.2%) and diet status (43.0%).

### 3.2 Association between healthy lifestyle with blood pressure measures

**Figure 1:**
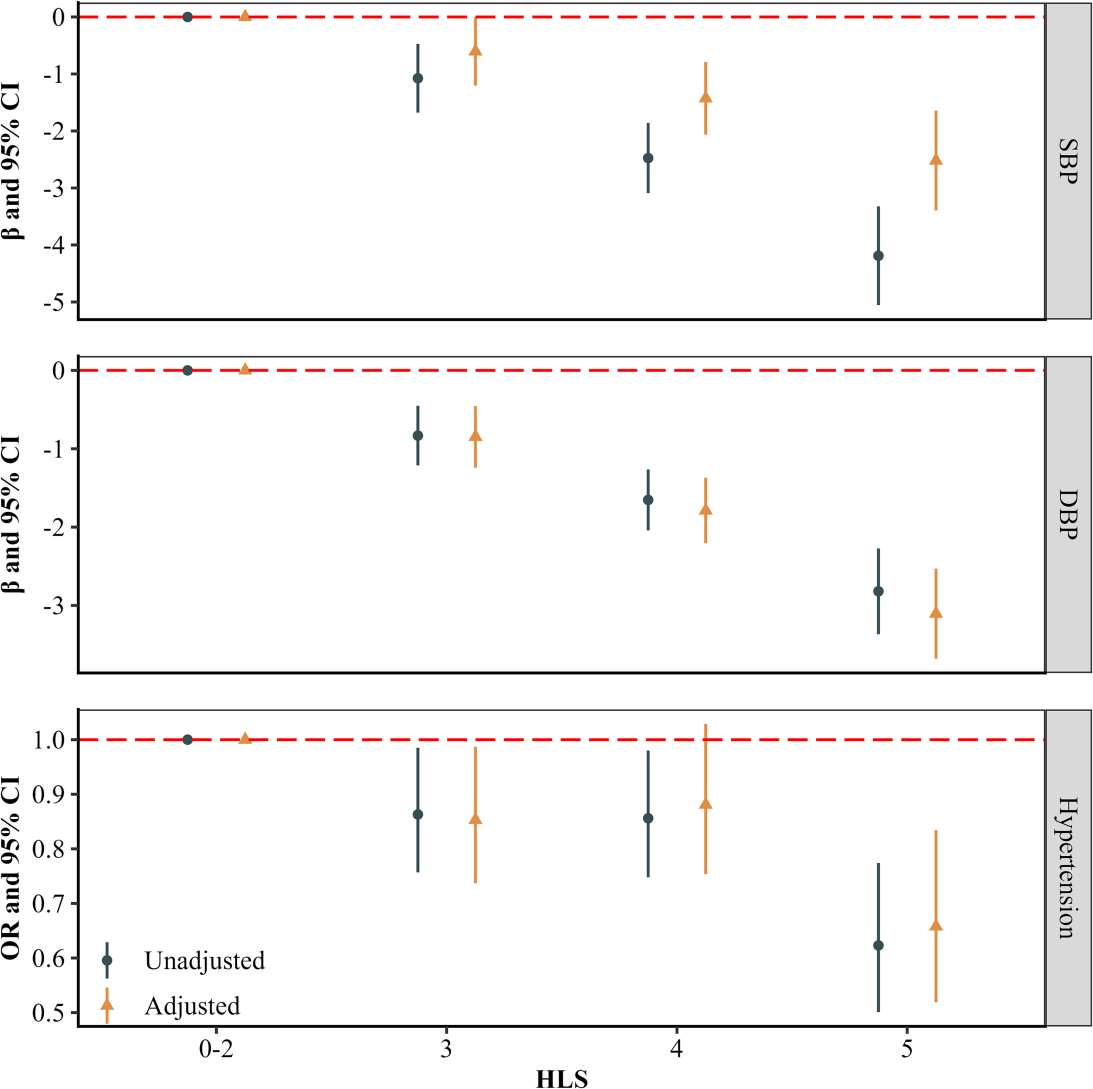
Associations of healthy lifestyles with blood pressure and hypertension (*n* = 16,454). The estimates were adjusted for age, gender, educational level, married status, per capita monthly income level, and history of chronic diseases (including type 2 diabetes mellitus, coronary heart diseases and stroke).

Figure 1 illustrates the associations between HLS and blood pressure measures. After multivariable adjustment, participants with HLS of 3, 4, and 5 had reductions in systolic blood pressure (SBP) of 0.604 mmHg (95% CI: 0.003, 1.204), 1.427 mmHg (95% CI: 0.791, 2.064), and 2.519 mmHg (95% CI: 1.644, 3.393), respectively, compared to those with HLS of 0–2 (all *p* < 0.05). Similarly, participants who scored 3, 4 and 5 showed reductions in DBP by 0.848 (0.455, 1.242), 1.788 (1.371, 2.205) mmHg and 3.105 (2.532, 3.678) mmHg, respectively (all *p* < 0.05). Furthermore, there was a significant trend indicating that higher HLS were associated with lower SBP and DBP levels (*P* for trend <0.001).

Supplementary Table S3 provides detailed estimates and 95% confidence intervals (95% *CIs*) for blood pressure measures associated with individual lifestyle factors. After multivariable adjustment, participants with a healthy lifestyle of smoking status displayed higher SBP and DBP levels compared to those with unhealthy smoking status (*p* < 0.05). Conversely, those with healthy drinking and BMI status had lower levels of SBP and DBP than those with unhealthy statuses (*p* < 0.05).

### 3.3 Association between healthy lifestyle with hypertension

**Figure 2:**
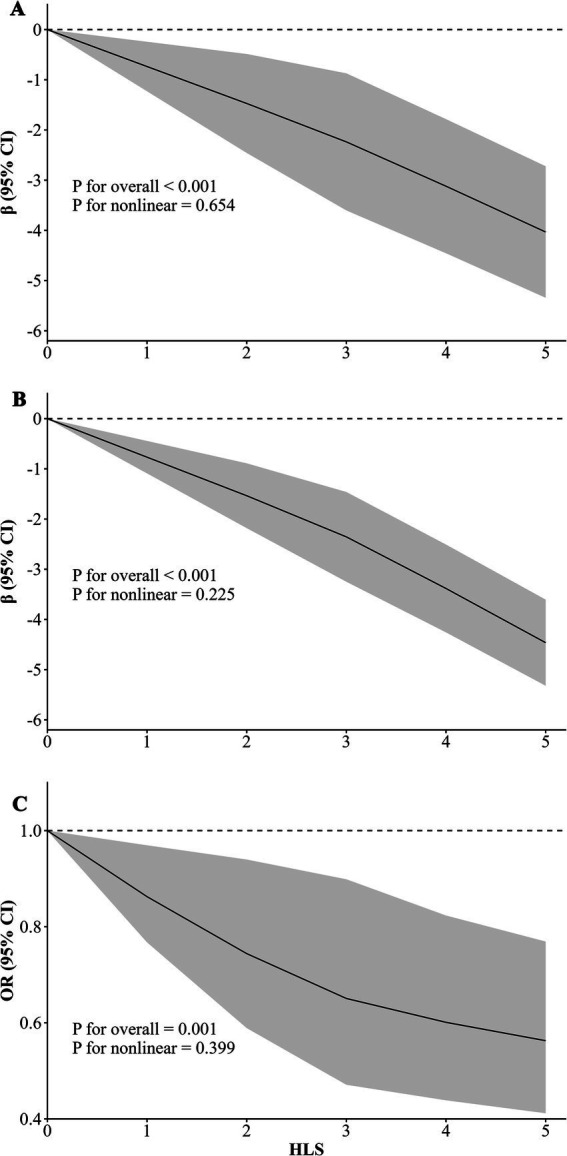
Dose–response relationship of healthy lifestyle score (HLS) with blood pressure and hypertension (*n* = 16,454). The graphs are labeled as follows: **(A)** systolic blood pressure (SBP), **(B)** diastolic blood pressure (DBP), and **(C)** hypertension risk (HTN). The estimates were adjusted for age, gender, educational level, marital status, per capita monthly income level, and history of chronic diseases (including type 2 diabetes mellitus, coronary heart diseases, and stroke).

Figure 1 presents the longitudinal associations between HLS and hypertension. After multivariable adjustment, participants with HLS of 3, 4, 5 had a reduced risk of hypertension (HLS = 3 OR = 0.853, 95% CI: 0.737, 0.987; HLS = 4 OR = 0.881, 95% CI: 0.754, 1.029; HLS = 5 OR = 0.658, 95% CI: 0.519, 0.834) compared with participants with HLS of 0–2. And a significant trend was observed, indicating a decreasing risk of hypertension with higher HLS (*P* for trend <0.05). Besides, Figure 2 displays the linear dose–response relationship between HLS with blood pressure measures and hypertension (*P* for non-linear >0.05). The results demonstrate that as HLS increased, both SBP and DBP levels, as well as the risk of hypertension declined (*p* < 0.001).

Supplementary Table S3 provides estimates and 95% confidence intervals (95% *CIs*) for blood pressure measures and hypertension associated with individual lifestyle factors. Participants with healthy alcohol consumption and BMI status were at lower risk of hypertension (*p* < 0.05).

### 3.4 Association between lifestyle change with blood pressure measures and hypertension

**Figure 3:**
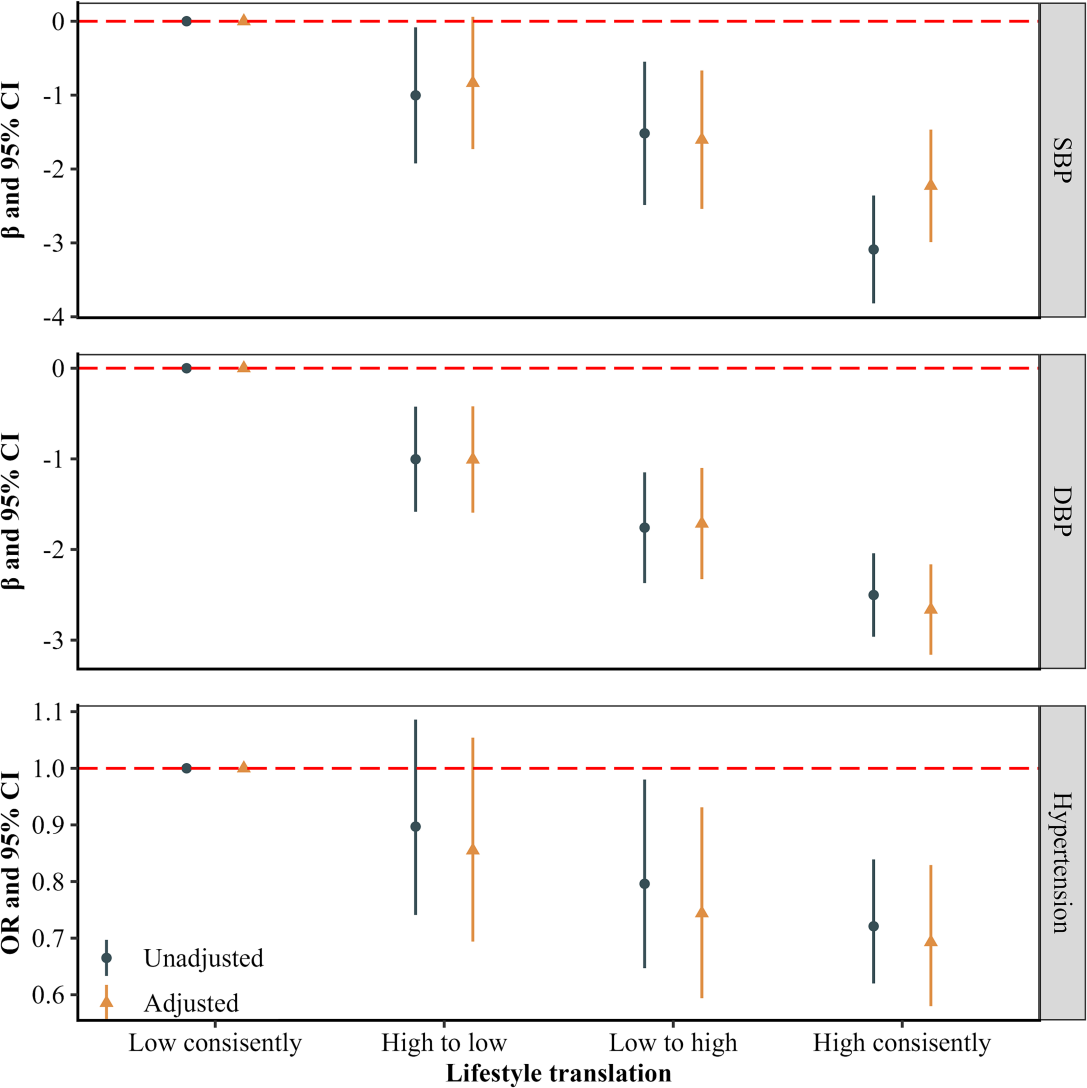
Associations of lifestyles change with blood pressure and hypertension (*n* = 16,454). The estimates were adjusted for age, gender, educational level, married status, per capita monthly income level, and history of chronic diseases (including type 2 diabetes mellitus, coronary heart diseases and stroke).

Figure 3 provides the associations between multiple lifestyle changes with blood pressure measures and hypertension. After multivariable adjustment, participants who changed from a high to low healthy lifestyle, from a low to high healthy lifestyle score, or maintained a consistently healthy lifestyle had decreased SBP by 0.835 (−0.058, 1.729) mmHg, 1.603 (0.668, 2.539) mmHg and 2.228 (1.467, 2.988) mmHg, respectively, compared to those with a consistently unhealthy lifestyle. Similarly, reductions in DBP were observed in these groups, with decrease of 1.006 (−0.420, 1.592) mmHg, 1.713 (1.100, 2.326) mmHg and 2.662 (2.163, 3.160) mmHg, respectively. Compared to participants with a consistently unhealthy lifestyle, those changing lifestyle scores from low to high had a lower risk of hypertension (OR = 0.744, 95% CI: 0.594, 0.931; *p* < 0.05). Participants who maintained a consistently healthy lifestyle also had a lower risk of hypertension (OR = 0.693, 95% CI: 0.580, 0.829; *p* < 0.05). However, participants who changed from high to low HLS did not show a significant difference in hypertension risk compared to those with a consistently unhealthy lifestyle (*p* > 0.05).

Supplementary Table S4 presents estimates and 95% confidence intervals (95% *CIs*) for blood pressure measures and hypertension associated with individual lifestyle changes. After multivariable adjustments, participants who transitioned from an unhealthy to a healthy BMI status experienced a greater decrease in SBP than those with a consistently unhealthy lifestyle. Additionally, participants who improved from unhealthy to healthy status in drinking and BMI displayed lower DBP compared to those with a consistently unhealthy lifestyle (*p* < 0.05). Moreover, participants who adopted healthier lifestyles in both physical activity and BMI status also demonstrated a lower risk of hypertension (*p* < 0.05).

### 3.5 Sensitivity analysis

Sensitivity analysis 1 shows that when using the weighted HLS for analysis, with one standard deviation increment of the weighted HLS, the SBP increased 2.485 (2.154, 2.816) mmHg, the DBP increased 2.386 (2.170, 2.602) mmHg, and the risk of hypertension increased 1.319 (1.220, 1.425), supporting the robustness of the main analysis (Supplementary Table S5). Sensitivity analysis 2 further adjusted for region, sensitivity analysis 3 excluding participants with hypertension occurrence within 1 year after the baseline survey, and sensitivity analysis 4 which excluded participants with coronary heart diseases or stroke in the baseline survey all found the results were consistent to the main analysis (Supplementary Table S5). Supplementary Table S6 indicates that among participants with hypertension but not using anti-hypertension medicine, a healthier lifestyle was similarly beneficial for DBP control as observed in those without hypertension. But for participants with hypertension who were using anti-hypertension medicine use, a higher HLS could not significantly lower blood pressure measures.

## 4 Discussion

The study found that adherence to a healthier lifestyle could lower the level of blood pressure and reduce the risk of hypertension in resource-limited distracts based on the cohort study. What’s more, changing from an unhealthy to healthy lifestyle was found to be effective in lowering blood pressure level and preventing hypertension.

This research evaluated the impact of healthy lifestyle on blood pressure and hypertension in a longitudinal study based on the Henan rural cohort study, suggesting that adopting a healthy lifestyle can lower blood pressure and prevent hypertension in resource-limited areas. Several studies have examined the association of HLS with hypertension. Hu et al. (31) found subjects with a higher HLS were associated with a lower risk of hypertension in the baseline of the Guangzhou Heart Study. But the cross-sectional design limits the ability to determine causal relationships. Several longitudinal studies have shown similar results. A Korean study found HLS was longitudinally associated with reduced risk of hypertension and suggested the combined lifestyles as a useful tool for hypertension prevention (32). Besides, based on the UK Biobank (UKB), the longitudinal associations of HLS with hypertension and cardiometabolic multimorbidity were revealed (33, 34). We conducted the longitudinal study in resource-limited distracts, providing evidence that adopting a healthy lifestyle is an effective strategy to prevent hypertension.

Our cohort study found that lifestyle changes could affect the risk of hypertension among rural adults. Previous studies have found the impacts of single lifestyle intervention on hypertension prevention, especially diet intervention (12). Recently, researchers explored the impact of multiple lifestyles changing on chronic non-infectious diseases, such as cardiovascular disease (CVD) (22, 35). However, the findings were inconsistent. A Chinese pragmatic cluster randomized controlled trial aiming to assess the effect of pharmaceutical and healthy lifestyle interventions on severe CVD events found that the interventions did not reduce serious CVD events over 36 months (762 and 874 severe CVD events in the intervention and control group, respectively) but SBP and DBP were lower in intervention group than those in control group. While a UKB study found participants with poor lifestyles consistently and changing healthy one to an unhealthy one were more likely to have ischemic heart diseases compared with those with healthy lifestyle consistently (35). We found participants with healthy lifestyle consistently could lower level of blood pressure and the risk of hypertension. Interestingly, we found changing lifestyle from unhealthy to healthy could lower blood pressure and prevent hypertension. The results could inform hypertension prevention guidelines by emphasizing the role of lifestyle interventions in resource-limited distracts.

This study has several strengths. It is a large-scale study that employed multivariable adjusted analyses to account for potential confounding factors and used restricted cubic splines to explore possible non-linear dose–response relationships between lifestyle factors and blood pressure measures. Additionally, multiple sensitivity analyses were conducted to ensure the robustness of the findings across different scenarios. However, some limitations should be acknowledged in this study. The information about lifestyle factors was obtained by a self-reported questionnaire, which may cause recall bias. However, the retest of questionnaire showed high intra-class correlation coefficients (ICCs = 0.841) (18). Although there was a high level of agreement between baseline and follow-up self-reports of smoking status, physical activity, and BMI (kappa coefficients ranging from 0.57 to 0.94), these data may still be influenced by social desirability bias or misclassification by participants, particularly in rural areas where the concept of a ‘healthy lifestyle’ may not be well understood, leading to potential misunderstandings or underreporting of unhealthy behaviors (36). Additionally, since the participants were obtained from rural areas in Henan province of China, extrapolating the study results to other population should be cautious. Finally, although several important confounders were considered, other unmeasured or unselected factors (such as genetic and environmental factors) may affect the results of this study. In general, further research is needed to explore the broader applicability of these findings across different populations and settings.

## 5 Conclusion

The present study found that adapting a healthier lifestyle could lower blood pressure and prevent hypertension in rural adults. Furthermore, changing lifestyles from unhealthy to healthy, or insisting healthy lifestyles could effectively control blood pressure and prevent hypertension. These findings provide evidence supporting lifestyle interventions for hypertension prevention in resource-limited areas.

## Data Availability

The raw data supporting the conclusions of this article will be made available by the authors, without undue reservation.
