# Supplementary material for: Healthy lifestyle and its change attenuated the risk of hypertension among rural population: evidence from a prospective cohort study
Source: Front Public Health. 2025 Feb 6;13:1529570. doi: 10.3389/fpubh.2025.1529570 (PMC11839643; doi:10.3389/fpubh.2025.1529570)

**Supplementary materials.**

**Supplementary table 1.** Composition and points of the diet quality score.

**Supplementary table 2.** Description and prevalence of the components of healthy lifestyle score.

**Supplementary table 3.** Association of single lifestyle with blood pressure measures and hypertension.

**Supplementary table 4.** Association of single lifestyle change with blood pressure measures and hypertension.

**Supplementary table 5.** Sensitivity analysis.

**Supplementary table 6.** Association of HLS with blood pressure measures stratified by hypertension and anti-hypertension medicine use.

**Supplementary figure 1.** Flow chart.

**Supplementary table 1.** Composition and points of the diet quality score, based on responses from food frequency questionnaire. (Ranging from 0 to 36)

| **Food group** | **Food frequency questionnaire responses** | | | | | **Total Max** |
| --- | --- | --- | --- | --- | --- | --- |
|  | **Once per day** | **Once**  **per week** | **Once**  **per month** | **Once**  **per year** | **Never** |  |
| **Red meat** | 0 | 1 | 2 | 3 | 4 | 4 |
| **Fish** | 4 | 3 | 2 | 1 | 0 | 4 |
| **Eggs** | 4 | 3 | 2 | 1 | 0 | 4 |
| **Dairy food** | 4 | 3 | 2 | 1 | 0 | 4 |
| **Been products** | 4 | 3 | 2 | 1 | 0 | 4 |
| **Nut** | 4 | 3 | 2 | 1 | 0 | 4 |
| **Whole grains** | 4 | 3 | 2 | 1 | 0 | 4 |
| **Fruits** | 4 | 3 | 2 | 1 | 0 | 4 |
| **Vegetables** | 4 | 3 | 2 | 1 | 0 | 4 |
| **Total** |  |  |  |  |  | 36 |

**Supplementary table 2.** Description and prevalence of the components of healthy lifestyle score (n = 16454).

| **Components** **of HLS** | **Score** | **Description** | **Prevalence, n (%)** | |
| --- | --- | --- | --- | --- |
|  |  |  | **Baseline** | **Follow-up** |
| **Smoking status** | 0 | Current smoking or quit smoking for illness | 3972 (24.1) | 3845 (23.4) |
|  | 1 | Never smoking or quit smoking for reasons other than illness | 12482 (75.9) | 12609 (76.6) |
| **Drinking status** | 0 | Excessive alcohol consumption (≥25 g/day for men, ≥15 g/day for women) | 1182 (7.2) | 1070 (6.5) |
|  | 1 | Limited Alcohol Consumption (<25 g/day for men, <15 g/day for women) | 15272 (92.8) | 15384 (93.5) |
| **Physical activity** | 0 | Low physical activity | 4636 (28.2) | 5979 (36.3) |
|  | 1 | Moderate or vigorous physical activity | 11818 (71.8) | 10475 (63.7) |
| **Diet status*** | 0 | Diet score < 22 | 9934 (60.4) | 9371 (57.0) |
|  | 1 | Diet score ≥ 22 | 6520 (39.6) | 7083 (43.0) |
| **BMI status** | 0 | Underweight, overweight or obesity (BMI<18.5 kg/m^2^ or BMI>23.9 kg/m^2^) | 8857 (53.8) | 9024 (54.8) |
|  | 1 | Normal weight (BMI:18.5-23.9 kg/m^2^) | 7597 (46.2) | 7430 (45.2) |

Abbreviation: BMI, body mass index; HLS, healthy lifestyle score.

*Diet status was evaluated by a diet quality score, which was computed based on the availability of data from food frequency questionnaire, ranging from 0 to 36.

**Supplementary table 3.** Association of single lifestyle with blood pressure measures and hypertension (n = 16454).

| **Lifestyle** | **SBP *β* (95% CI)** | |  | **DBP *β* (95% CI)** | |  | | **HTN OR (95% CI)** | |
| --- | --- | --- | --- | --- | --- | --- | --- | --- | --- |
|  | **Crude model** | **Adjusted model** |  | **Crude model** | **Adjusted model** |  | **Crude model** | | **Adjusted model** |
| **Smoking status** |  |  |  |  |  |  |  | |  |
| Current smoking or quit smoking for illness | Reference | Reference |  | Reference | Reference |  | Reference | | Reference |
| Never smoking or quit smoking for reasons other than illness | -0.872 (-1.384, -0.360) | 1.595 (0.876, 2.315) |  | -0.079 (-0.402, 0.244) | 0.835 (0.362, 1.308) |  | 0.999 (0.884, 1.129) | | 1.092 (0.914, 1.304) |
| **Drinking status** |  |  |  |  |  |  |  | |  |
| Excessive alcohol consumption | Reference | Reference |  | Reference | Reference |  | Reference | | Reference |
| Limited alcohol consumption | -2.833 (-3.680, -1.986) | -2.325 (-3.186, -1.465) |  | -2.242 (-2.776, -1.707) | -1.998 (-2.563, -1.433) |  | 0.711 (0.593, 0.852) | | 0.671 (0.551, 0.817) |
| **Diet status** |  |  |  |  |  |  |  | |  |
| Diet score < 22 | Reference | Reference |  | Reference | Reference |  | Reference | | Reference |
| Diet score ≥ 22 | -0.819 (-1.267, -0.372) | 0.318 (-0.120, 0.755) |  | 0.555 (0.272, 0.837) | 0.243 (-0.045, 0.531) |  | 0.989 (0.889, 1.101) | | 1.081 (0.969, 1.207) |
| **Physical activity** |  |  |  |  |  |  |  | |  |
| Low physical activity | Reference | Reference |  | Reference | Reference |  | Reference | | Reference |
| Moderate or vigorous physical activity | -0.455 (-0.941, 0.032) | -0.055 (-0.522, 0.411) |  | -0.282 (-0.589, 0.026) | -0.278 (-0.585, 0.029) |  | 0.937 (0.836, 1.052) | | 0.986 (0.878, 1.108) |
| **BMI status** |  |  |  |  |  |  |  | |  |
| Underweight, overweight or obesity | Reference | Reference |  | Reference | Reference |  | Reference | | Reference |
| Normal weight | -2.430 (-2.868, -1.993) | -2.946 (-3.365, -2.526) |  | -3.032 (-3.306, -2.759) | -2.984 (-3.257, -2.710) |  | 0.760 (0.683, 0.846) | | 0.722 (0.648, 0.805) |

Abbreviation: BMI: body mass index; CI: confidence interval; DBP: diastolic blood pressure; HTN: hypertension; OR: odds ratio; SBP: systolic blood pressure.

Adjusted model was adjusted for age, gender, educational level, married status, per capita monthly income level, and history of chronic diseases (including type 2 diabetes mellitus, coronary heart diseases and stroke).

**Supplementary table 4.** Association of single lifestyle change with blood pressure measures and hypertension (n = 16454).

| **Lifestyle change** | **SBP** β **(95% CI)** | |  | **DBP** β **(95% CI)** | |  | **HTN** OR **(95% CI)** | |
| --- | --- | --- | --- | --- | --- | --- | --- | --- |
|  | Crude model | Adjusted model |  | Crude model | Adjusted model |  | Crude model | Adjusted model |
| Smoking status change |  |  |  |  |  |  |  |  |
| Low to low | Reference | Reference |  | Reference | Reference |  | Reference | Reference |
| High to low | 0.680 (-0.768, 2.129) | 0.188 (-1.193, 1.57) |  | -0.372 (-1.287, 0.543) | -0.238 (-1.147, 0.67) |  | 0.798 (0.546, 1.164) | 0.757 (0.518, 1.107) |
| Low to high | 1.922 (0.631, 3.213) | 0.96 (-0.275, 2.196) |  | 0.288 (-0.527, 1.103) | 0.551 (-0.261, 1.364) |  | 1.091 (0.807, 1.475) | 0.996 (0.735, 1.349) |
| High to high | -0.651 (-1.195, -0.107) | 2.101 (1.31, 2.893) |  | -0.028 (-0.371, 0.316) | 1.188 (0.668, 1.709) |  | 1.019 (0.894, 1.161) | 1.179 (0.972, 1.429) |
| P for trend | 0.006 | <0.001 |  | 0.955 | <0.001 |  | 0.635 | 0.107 |
| Drinking status change |  |  |  |  |  |  |  |  |
| Low to low | Reference | Reference |  | Reference | Reference |  | Reference | Reference |
| High to low | -2.076 (-3.794, -0.359) | -2.295 (-3.934, -0.656) |  | -1.878 (-2.960, -0.795) | -1.812 (-2.888, -0.735) |  | 0.878 (0.602, 1.281) | 0.844 (0.577, 1.236) |
| Low to high | -0.4 (-2.035, 1.234) | -0.736 (-2.297, 0.824) |  | -1.778 (-2.808, -0.748) | -1.652 (-2.676, -0.627) |  | 1.061 (0.750, 1.500) | 1.007 (0.710, 1.429) |
| High to high | -3.076 (-4.283, -1.870) | -2.757 (-3.954, -1.559) |  | -3.221 (-3.981, -2.46) | -2.977 (-3.764, -2.191) |  | 0.729 (0.562, 0.945) | 0.657 (0.499, 0.866) |
| P for trend | <0.001 | <0.001 |  | <0.001 | <0.001 |  | 0.001 | <0.001 |
| Diet status change |  |  |  |  |  |  |  |  |
| Low to low | Reference | Reference |  | Reference | Reference |  | Reference | Reference |
| High to low | -0.144 (-0.789, 0.501) | 0.573 (-0.047, 1.192) |  | 0.543 (0.135, 0.950) | 0.325 (-0.082, 0.732) |  | 1.098 (0.946, 1.275) | 1.168 (1.004, 1.359) |
| Low to high | -0.706 (-1.309, -0.103) | -0.082 (-0.661, 0.496) |  | 0.132 (-0.249, 0.513) | -0.056 (-0.437, 0.324) |  | 0.969 (0.838, 1.119) | 1.016 (0.878, 1.176) |
| High to high | -1.657 (-2.222, -1.091) | 0.083 (-0.477, 0.644) |  | 0.634 (0.277, 0.991) | 0.15 (-0.218, 0.519) |  | 0.900 (0.784, 1.034) | 1.026 (0.888, 1.184) |
| P for trend | <0.001 | 0.974 |  | 0.003 | 0.668 |  | 0.120 | 0.849 |
| Physical activity status change |  |  |  |  |  |  |  |  |
| Low to low | Reference | Reference |  | Reference | Reference |  | Reference | Reference |
| High to low | -0.771 (-1.542, 0) | -0.074 (-0.812, 0.664) |  | -0.122 (-0.609, 0.364) | -0.172 (-0.657, 0.313) |  | 0.922 (0.777, 1.093) | 0.986 (0.829, 1.172) |
| Low to high | -1.314 (-2.147, -0.480) | -0.343 (-1.14, 0.454) |  | -0.069 (-0.595, 0.457) | -0.227 (-0.751, 0.297) |  | 0.723 (0.596, 0.878) | 0.788 (0.648, 0.959) |
| High to high | -1.425 (-2.131, -0.720) | -0.344 (-1.022, 0.334) |  | -0.423 (-0.868, 0.023) | -0.53 (-0.975, -0.084) |  | 0.720 (0.614, 0.844) | 0.806 (0.686, 0.948) |
| P for trend | <0.001 | 0.209 |  | 0.025 | 0.006 |  | <0.001 | 0.001 |
| BMI status change |  |  |  |  |  |  |  |  |
| Low to low | Reference | Reference |  | Reference | Reference |  | Reference | Reference |
| High to low | -0.793 (-1.627, 0.041) | -0.893 (-1.687, -0.099) |  | -1.270 (-1.788, -0.751) | -1.283 (-1.799, -0.766) |  | 1.070 (0.890, 1.287) | 1.056 (0.877, 1.273) |
| Low to high | -3.439 (-4.324, -2.554) | -4.107 (-4.949, -3.264) |  | -3.412 (-3.962, -2.862) | -3.252 (-3.801, -2.704) |  | 0.713 (0.569, 0.893) | 0.659 (0.525, 0.827) |
| High to high | -3.308 (-3.782, -2.833) | -4.018 (-4.472, -3.564) |  | -3.932 (-4.227, -3.637) | -3.849 (-4.144, -3.554) |  | 0.664 (0.590, 0.747) | 0.616 (0.547, 0.695) |
| P for trend | <0.001 | <0.001 |  | <0.001 | <0.001 |  | <0.001 | <0.001 |

Abbreviation: BMI: body mass index; CI: confidence interval; DBP: diastolic blood pressure; HLS: healthy lifestyle score; HTN: hypertension; OR: odds ratio; SBP: systolic blood pressure.

Adjusted model was adjusted for age, gender, educational level, married status, per capita monthly income level, and history of chronic diseases (including type 2 diabetes mellitus, coronary heart diseases, and stroke).

**Supplementary table 5.** Sensitivity analysis.

| **HLS** | **SBP *β* (95% CI)** |  | **DBP *β* (95% *CI*)** |  | **HTN OR (95% *CI*)** |
| --- | --- | --- | --- | --- | --- |
| **Sensitivity analysis 1** | |  |  |  |  |
| Weighted HLS | 2.485 (2.154, 2.816) |  | 2.386 (2.170, 2.602) |  | 1.358 (1.251, 1.475) |
| **Sensitivity analysis 2** | |  |  |  |  |
| 0-2 | Reference |  | Reference |  | Reference |
| 3 | -0.59 (-1.188, 0.008) |  | -0.863 (-1.253, -0.473) |  | 0.847 (0.732, 0.981) |
| 4 | -1.508 (-2.144, -0.872) |  | -1.954 (-2.368, -1.539) |  | 0.854 (0.730, 0.998) |
| 5 | -2.711 (-3.586, -1.836) |  | -3.406 (-3.976, -2.835) |  | 0.623 (0.491, 0.791) |
| *P* for trend | <0.001 |  | <0.001 |  | <0.001 |
| **Sensitivity analysis 3** | |  |  |  |  |
| 0-2 | Reference |  | Reference |  | Reference |
| 3 | -0.572 (-1.171, 0.028) |  | -0.828 (-1.222, -0.435) |  | 0.859 (0.738, 1.000) |
| 4 | -1.416 (-2.052, -0.781) |  | -1.770 (-2.187, -1.353) |  | 0.914 (0.779, 1.074) |
| 5 | -2.489 (-3.361, -1.617) |  | -3.078 (-3.650, -2.505) |  | 0.695 (0.545, 0.887) |
| *P* for trend | <0.001 |  | <0.001 |  | 0.038 |
| **Sensitivity analysis 4** | |  |  |  |  |
| 0-2 | Reference |  | Reference |  | Reference |
| 3 | -0.604 (-1.204, -0.003) |  | -0.848 (-1.242, -0.455) |  | 0.834 (0.715, 0.972) |
| 4 | -1.427 (-2.064, -0.791) |  | -1.788 (-2.205, -1.371) |  | 0.871 (0.740, 1.024) |
| 5 | -2.519 (-3.393, -1.644) |  | -3.105 (-3.678, -2.532) |  | 0.629 (0.490, 0.808) |
| *P* for trend | <0.001 |  | <0.001 |  | 0.004 |

Abbreviation: CI: confidence interval; DBP: diastolic blood pressure; HLS: healthy lifestyle score; HTN: hypertension; OR: odds ratio; SBP: systolic blood pressure.

Sensitivity analysis 1 was adjusted for age, gender, educational level, married status, per capita monthly income level, and history of chronic diseases (including type 2 diabetes mellitus, coronary heart diseases, and stroke), and used the weighted HLS for analysis.

Sensitivity analysis 2 was adjusted for age, gender, educational level, married status, per capita monthly income level, history of chronic diseases (including type 2 diabetes mellitus, coronary heart diseases, and stroke), and region.

Sensitivity analysis 3 was adjusted for age, gender, educational level, married status, per capita monthly income level, and history of chronic diseases (including type 2 diabetes mellitus, coronary heart diseases, and stroke), and excluding participants with hypertension occurrence in one year after the baseline survey.

Sensitivity analysis 4 was adjusted for age, gender, educational level, married status, per capita monthly income level, and history of type 2 diabetes mellitus, and excluding participants with coronary heart diseases or stroke in the baseline survey.

**Supplementary table 6.** Association of HLS with blood pressure measures stratified by hypertension and anti-hypertension medicine use.

| **HLS** | **SBP *β* (95% *CI*)** | |  | **DBP *β* (95% *CI*)** | |
| --- | --- | --- | --- | --- | --- |
|  | **Crude model** | **Adjusted model** |  | **Crude model** | **Adjusted model ^a^** |
| **HTN=0** |  |  |  |  |  |
| 0-2 | Reference | Reference |  | Reference | Reference |
| 3 | -0.939 (-1.462, -0.417) | -0.300 (-0.822, 0.221) |  | -0.558 (-0.913, -0.204) | -0.627 (-0.992, -0.261) |
| 4 | -2.583 (-3.117, -2.049) | -1.412 (-1.966, -0.859) |  | -1.437 (-1.799, -1.074) | -1.642 (-2.030, -1.254) |
| 5 | -3.787 (-4.531, -3.043) | -2.093 (-2.846, -1.339) |  | -2.288 (-2.793, -1.783) | -2.672 (-3.200, -2.144) |
| *P* for trend | <0.001 | <0.001 |  | <0.001 | <0.001 |
| **HTN=1 & Medicine use = 1** | |  |  |  |  |
| 0-2 | Reference | Reference |  | Reference | Reference |
| 3 | -0.687 (-4.522, 3.149) | -1.295 (-5.239, 2.649) |  | 0.936 (-1.526, 3.397) | 0.944 (-1.542, 3.430) |
| 4 | 0.668 (-3.458, 4.795) | 0.982 (-3.422, 5.386) |  | -0.502 (-3.15, 2.146) | -0.521 (-3.297, 2.254) |
| 5 | -3.182 (-11.389, 5.026) | -1.611 (-9.766, 6.545) |  | -0.869 (-6.136, 4.397) | -0.648 (-5.789, 4.492) |
| *P* for trend | 0.958 | 0.773 |  | 0.566 | 0.568 |
| **HTN=1 & Medicine use = 0** | |  |  |  |  |
| 0-2 | Reference | Reference |  | Reference | Reference |
| 3 | 1.893 (0.346, 3.441) | 0.292 (-1.196, 1.781) |  | 0.936 (-1.526, 3.397) | -0.648 (-1.871, 0.576) |
| 4 | 1.311 (-0.249, 2.872) | -0.019 (-1.561, 1.524) |  | -0.502 (-3.15, 2.146) | -1.410 (-2.677, -0.142) |
| 5 | 2.097 (-0.295, 4.489) | 0.703 (-1.558, 2.963) |  | -0.869 (-6.136, 4.397) | -2.841 (-4.699, -0.984) |
| *P* for trend | 0.108 | 0.816 |  | <0.001 | 0.001 |

^*^: *P* < 0.05.

Abbreviation: CI: confidence interval; DBP: diastolic blood pressure; HLS: healthy lifestyle score; HTN: hypertension; SBP: systolic blood pressure.

Adjusted model was adjusted for age, gender, educational level, married status, per capita monthly income level, and history of chronic diseases (including type 2 diabetes mellitus, coronary heart diseases, and stroke).

**Supplementary figure 1. Flow chart.**


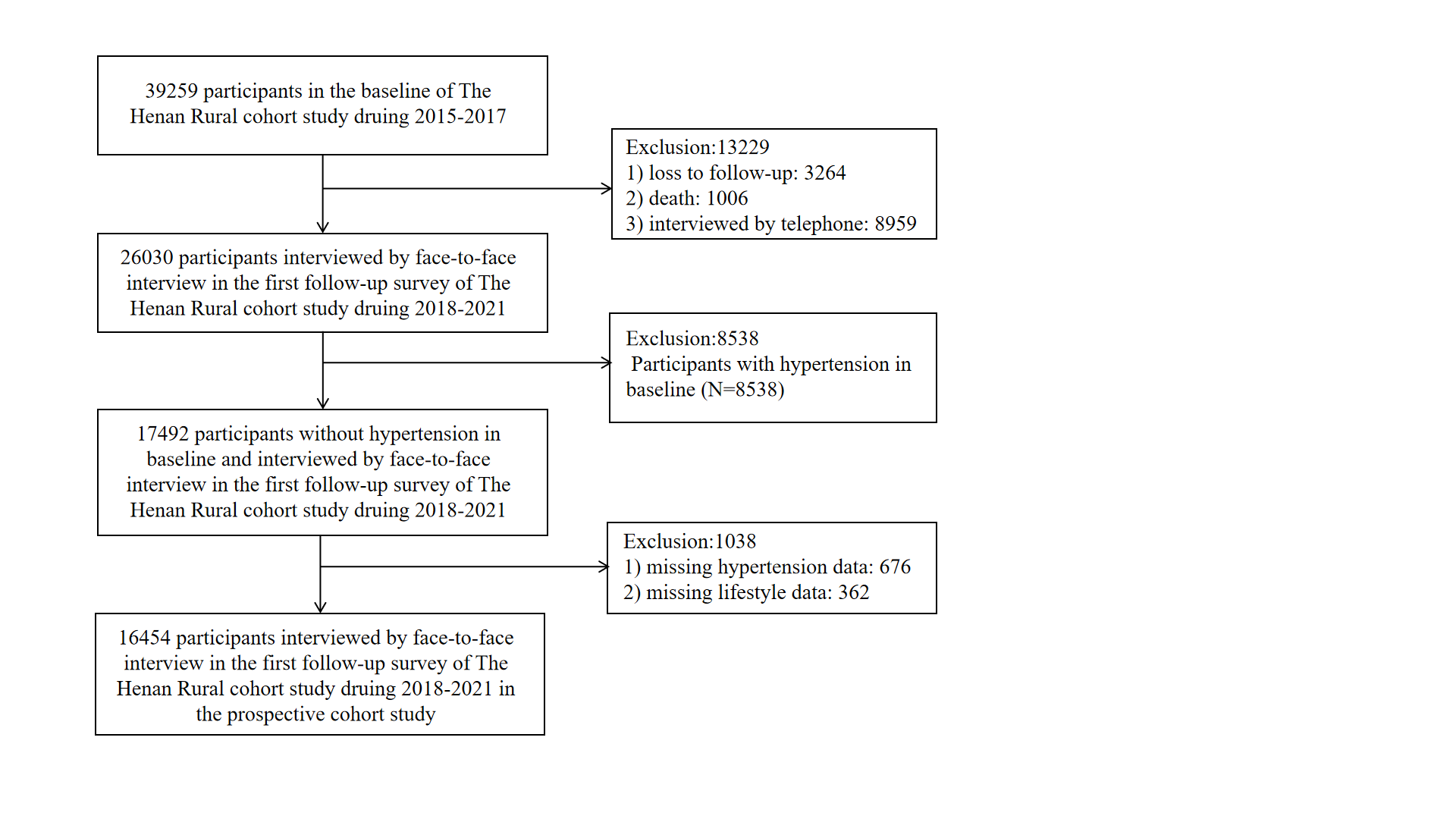

Supplement: Supplementary file 1 [file Data_Sheet_1.docx]
